# Radiation-Induced Esophagitis in Non-Small-Cell Lung Cancer Patients: Voxel-Based Analysis and NTCP Modeling

**DOI:** 10.3390/cancers14071833

**Published:** 2022-04-05

**Authors:** Serena Monti, Ting Xu, Radhe Mohan, Zhongxing Liao, Giuseppe Palma, Laura Cella

**Affiliations:** 1 Institute of Biostructures and Bioimaging, National Research Council, 80145 Napoli, Italy; serena.monti@ibb.cnr.it; 2 Department of Radiation Oncology, The University of Texas MD Anderson Cancer Center, Houston, TX 77030, USA; txu@mdanderson.org (T.X.); zliao@mdanderson.org (Z.L.); 3 Department of Radiation Physics, The University of Texas MD Anderson Cancer Center, Houston, TX 77030, USA; rmohan@mdanderson.org; 4 Institute of Nanotechnology, National Research Council, 73100 Lecce, Italy

**Keywords:** lung cancer, radiation-induced esophagitis, IMRT, proton therapy, voxel-based analysis, NTCP

## Abstract

**Simple Summary:**

Radiation-induced esophagitis (RE) is a common dose-limiting complication associated with concurrent chemoradiation therapy for Non-Small-Cell Lung Cancer (NSCLC), and a wide range of esophageal dosimetric parameters have been described as predictive of RE. In this study, we characterize the risk of RE for NSCLC patients enrolled in a prospective trial comparing intensity-modulated RT versus passive scattering proton therapy for locally advanced NSCLC. Dose patterns associated with RE were analyzed by applying voxel-based analysis approaches, and predictive models for RE were finally investigated. Two predictive models for acute RE with good cross-validated predictive performances and discrimination capability were developed (thoracic esophageal model: ROC-AUC = 0.73; whole esophagus model: ROC-AUC = 0.70).

**Abstract:**

The aim of our study is to characterize the risk of radiation-induced esophagitis (RE) in a cohort of Non-Small-Cell Lung Cancer (NSCLC) patients treated with concurrent chemotherapy and photon/proton therapy. For each patient, the RE was graded according to the CTCAE v.3. The esophageal dose-volume histograms (DVHs) were extracted. Voxel-based analyses (VBAs) were performed to assess the spatial patterns of the dose differences between patients with and without RE of grade ≥ 2. Two hierarchical NTCP models were developed by multivariable stepwise logistic regression based on non-dosimetric factors and on the DVH metrics for the whole esophagus and its anatomical subsites identified by the VBA. In the 173 analyzed patients, 76 (44%) developed RE of grade ≥ 2 at a median follow-up time of 31 days. The VBA identified regions of significant association between dose and RE in a region encompassing the thoracic esophagus. We developed two NTCP models, including the RT modality and a dosimetric factor: V_55Gy_ for the model related to the whole esophagus, and the mean dose for the model designed on the thoracic esophagus. The cross-validated performance showed good predictions for both models (ROC-AUC of 0.70 and 0.73, respectively). The only slight improvement provided by the analysis of the thoracic esophageal subsites might be due to the relevant sparing of cervical and lower thoracic esophagus in the analyzed cohort. Further studies on larger cohorts and a more heterogeneous set of dose distributions are needed to validate these preliminary findings and shed further light on the spatial patterns of RE development.

## 1. Introduction

The most recent technological developments have significantly improved the accuracy of thoracic radiation therapy (RT) for lung cancer, allowing for the delivery of highly conformal dose distributions [1,2]. Nevertheless, radiation-induced esophagitis (RE) remains a common dose-limiting toxicity associated with RT for Non-Small-Cell Lung Cancer (NSCLC) treated with chemotherapy (CHT). Acute RE generally develops in the first 2 or 3 weeks after the start of RT and potentially affects quality of life and, in the most severe cases, may result in treatment interruption [3,4].

Our ability to identify patients at an increased risk of developing RE is, however, still limited. Several clinical factors have been described as associated with RE; in particular, concurrent CHT may increase the risk of RE occurrence [5]. A wide range of esophageal dosimetric factors have also been reported as predictive for RE [6] and conflicting results can be found in the literature [7]. They describe different radiobiologies for the esophagus, consistent either with a serial behavior or with a near-parallel, or even with a full-parallel behavior [8,9,10,11,12]. A radiobiological sensitivity difference between the proximal and distal portions of the esophagus has also been evaluated in preclinical research [13], while clinical studies suggested that the irradiation of the upper portions of the esophagus may represents a risk factor for the occurrence of acute RE, compared to other segments [14,15]. In the above-cited studies, the potential differential spatial properties of the esophagus were investigated by standard dosimetric analyses, which rely on an organ-based approach.

Nowadays, we can exploit a new method for investigating such organ characteristics, the so-called voxel-based analysis (VBA), which has proven to be a powerful tool to underpin potential regional dependency of organ radiosensitivity [16,17,18]. In the most recent years, indeed, this new emerging methodology allowed for the preservation of the full spatial information of the planned dose from each patient. Thereby, no assumptions are made regarding the predefined anatomical regions; instead, the dose in every voxel across many patients is analyzed against a given radiation-induced outcome. This process identifies the anatomical regions in which dose deposition is most strongly correlated with these outcomes and, therefore, can better define the anatomy driving them [19,20,21,22,23,24,25].

The aim of the present study is to characterize the risk of RE for NSCLC patients enrolled in a prospective trial comparing intensity-modulated RT (IMRT) versus passive scattering proton therapy (PSPT) for locally advanced NSCLC. The dose patterns associated with RE are analyzed by applying VBA approaches, and the predictive models for RE are finally investigated.

## 2. Materials and Methods

### 2.1. Patient Cohort

We retrospectively analyzed locally advanced NSCLC patients who were treated at the University of Texas MD Anderson Cancer in a prospective randomized trial (NCT00915005). Patients were treated with IMRT or PSPT at a prescribed dose of 66 or 74 Gy in a conventional daily 2 Gy fractionation (relative biological effectiveness—RBE—was set to 1.1 for protons) with concurrent chemotherapy (CHT), according to an institutional review board-approved protocol (2008-0133). The different levels of doses of 66 or 74 Gy (RBE) were prescribed, depending on normal tissue constraint’s compliance. The trial protocol, patient and treatment characteristics were previously described [26,27].

Patients were clinically evaluated once a week during CHT-RT for an assessment of adverse events, including esophagitis. Patient were then evaluated with images studies, blood works and seen by a physician in follow-up clinic at 6–8 weeks, 3–4 month for the first 3 years after CHT-RT, every 6 months for years 4–5, and yearly thereafter. RE was graded according to the National Cancer Institute’s Common Terminology Criteria for Adverse Events v. 3. The time to RE was considered from the start of RT to the date of the first-documented RE. Patients who did not develop RE were censored at death or at the last follow up. The endpoint of the following analyses was acute grade 2 (G2) or higher RE, hereafter referred to as RE.

For the single time-point analysis, the relationships between non-dosimetric factors and binary RE were tested by Pearson’s χ^2^ test and by the Mann–Whitney *U* test, when appropriate. For actuarial analysis, the correlation between the non-dosimetric variables and RE were tested with Cox proportional hazard models. Statistical tests were performed with SPSS 27.0 statistical software (SPSS Inc., Chicago, IL, USA).

From the described patient cohort, subjects with complete planning computed tomography (CT) scans and dose maps were selected for further analysis.

All PSPT patients were planned on the Eclipse (Varian Medical Systems, Palo Alto, CA, USA) proton treatment planning system using the algorithms described in [28] and subsequent improvements. All IMRT patients were planned on the Pinnacle treatment planning system (Philips, Radiation Oncology Systems, Fitchburg, WI, USA) using the collapsed cone convolution superposition dose algorithm. All dose maps were obtained with a dose grid size of 2.0 × 2.0 × 2.5 mm^3^, exported from treatment planning systems as DICOM RT files and converted for MATLAB (The MathWorks, Inc., Natick, MA, USA) by using CERR [29].

### 2.2. Dose-Volume Histogram Analysis

The esophageal dose-volume histograms (DVHs) were extracted and the average DVHs for patients with and without RE were compared. Similarly, the average DVHs for PSPT or IMRT treatment were compared to identify the potential dosimetric significant differences resulting from the RT modality. The significance of the DVH differences between the groups of patients was computed according to a non-parametric maximum-*T* permutation test that accounts for multiple comparisons [23].

### 2.3. Voxel-Based Analyses

Dose maps were preliminarily converted into a biologically effective dose (BED) using an alpha–beta ratio of 10 Gy for acute reacting tissues [8,9].

A voxel-based analysis (VBA) was performed to evaluate the different dose patterns between patients with and without RE. Details of the whole VBA pipeline are described elsewhere [30,31]. Briefly, planning CTs and BED maps were spatially normalized to a common anatomical reference (digital phantom XCAT, [32]) after masking the gross tumor volume [33] and by using a B-spline elastic image registration algorithm [34]. Registration accuracy is confirmed in previous studies based on the same cohort of patients [23]. The generalized linear model (GLM) was then designed to include dose maps and each confounding variable selected by a multivariable stepwise logistic regression method applied on a set of preselected variables (the complete set is reported in Table 1) [35]. A non-parametric permutation test of the maximum threshold-free cluster-enhanced statistic accounting for multiple comparisons was performed and the significance *p*-maps were obtained.

Additionally, an actuarial VBA was performed in relation to RE, with the inclusion of nuisance variables selected by a stepwise Cox regression analysis [23].

Using the common radiological landmarks described in [36], the esophagus was segmented into its four subsites on the XCAT phantom as follows: (1) the cervical esophagus from the cricoid cartilage to the sternal notch plane; (2) the upper thoracic esophagus from the sternal notch plane to the lower edge of the azygos vein; (3) the middle thoracic esophagus from the lower edge of the azygos vein to the lower pulmonary veins; and (4) the lower thoracic esophagus from the lower pulmonary veins to the gastroesophageal junction.

The differential histograms of the relative volume associated with a given *p*-value were generated for the different subsites.

The segmented esophageal subsites were then identified in the patients’ native space (i.e., planning CTs): each original esophageal planning structure was divided into four subsites according to the cranio-caudal coordinates of the back-propagated centers of mass of the three boundaries in the XCAT phantom space.

The DVHs for the subsites of interest were subsequently extracted.

### 2.4. Normal Tissue Complication Probability (NTCP) Modeling

In order to evaluate the impact of non-dosimetric and dosimetric factors on RE, the multivariable stepwise logistic regression method for NTCP modeling was applied [37]. The following dose metrics were extracted from DVHs for modeling: the relative volume receiving at least *x* dose (V*_x_*) in steps of 5 Gy, the near maximum dose (D_2%_) and the mean dose (D_mean_).

In the multivariable analysis, we only included the variables highly correlated with RE (*p* < 0.1 at the univariable analysis) that were not collinear (correlation |R_s_| < 0.70) with those variables more correlated with RE (variable selection step) [38,39,40]. Then, the actual NTCP models were built through a hierarchical approach [41]. In the first hierarchical block, a stepwise logistic regression was performed on the non-dosimetric variables and DVH metrics identified by the variable selection. Then, in the second block, a new logistic regression tested if the prediction improvements obtained by adding the RT modality predictor were statistically significant, thus possibly updating the first block model. The Leave-One-Out (LOO) method was applied to cross validate the models [42].

The area under the receiver operating characteristic (ROC) curve (AUC) was used to evaluate the model performance. Calibration plots were also generated to assess the agreement between the observed outcome and cross-validated prediction [43].

## 3. Results

We identified 202 patients; 127 (63%) were treated with IMRT and 75 (37%) with PSPT. The crude rate of RE was 45% (91 of 202). Twenty-six out of 91 (29%) patients developed grade 3 toxicity, while there were no cases of grades 4 or 5. The median time to RE was 31 days (range: [7, 90] days).

Of the 202 patients, 29 had incomplete or corrupted CT/dose info and were excluded from the analysis. The incidence of RE in the 173 analyzed patients was 44% (76/173), which was comparable with that observed in the full cohort. All patient characteristics of the full and reduced cohorts were comparable and are reported in Supplementary Table S1.

A single time-point analysis revealed that none of the clinical factors significantly correlated with RE. In the Cox analysis, RE was significantly higher in patients receiving induction CHT (Table 1 and Supplementary Figure S1a). The crude incidence of RE as well as the cumulative incidence (Table 1 and Supplementary Figure S1b) differed significantly between the RT modalities with a higher incidence in the PSPT group.

**Figure 1:**
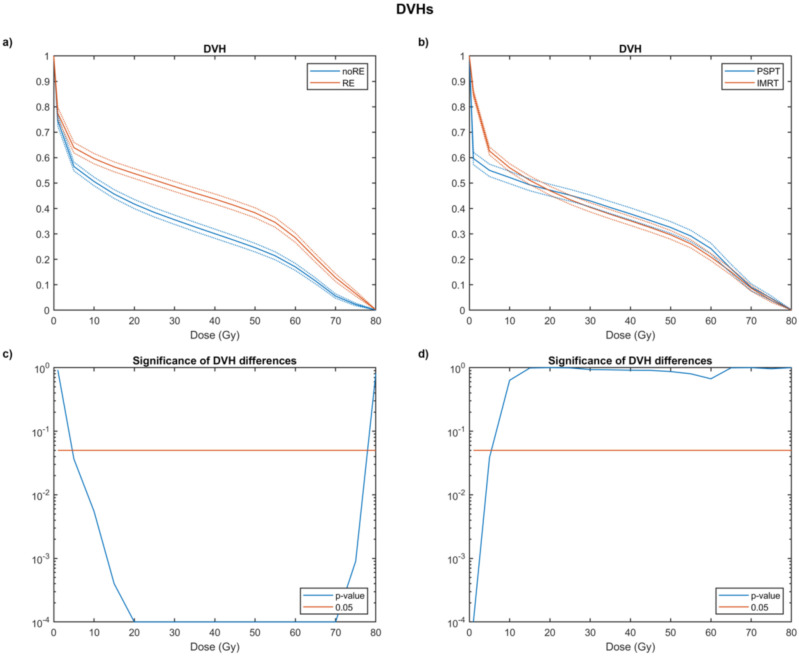
Average esophageal dose volume histograms (DVHs) ± SEM (standard error of the mean) in patients who developed radiation-induced esophagitis (RE) of grade ≥ 2 and not (**a**); average esophageal DVHs ± SEM (standard error of the mean) in patients treated with Intensity Modulated Radiation Therapy (IMRT) and Passive Scattering Proton Therapy (PSPT) (**b**). The SEM is plotted as dashed lines. Semi-logarithmic plot of the observed significance level estimated by a non-parametric maximum-*T* permutation test between DVH values for RE and unaffected patients (**c**); semi-logarithmic plot of the observed significance level estimated by a non-parametric maximum-*T* permutation test between DVH values for IMRT and PSPT (**d**).

The average DVHs of patients with and without RE showed a significant separation between the two curves starting from a dose value of 5 Gy (Figure 1a,c). The DVH analysis of the esophagus in patients stratified by treatment modality (Figure 1b,d) revealed that PSPT significantly reduced the low-dose (<5 Gy) irradiated volume. No significant differences between PSPT and IMRT were instead highlighted for the dose values greater than 5 Gy.

**Figure 2:**
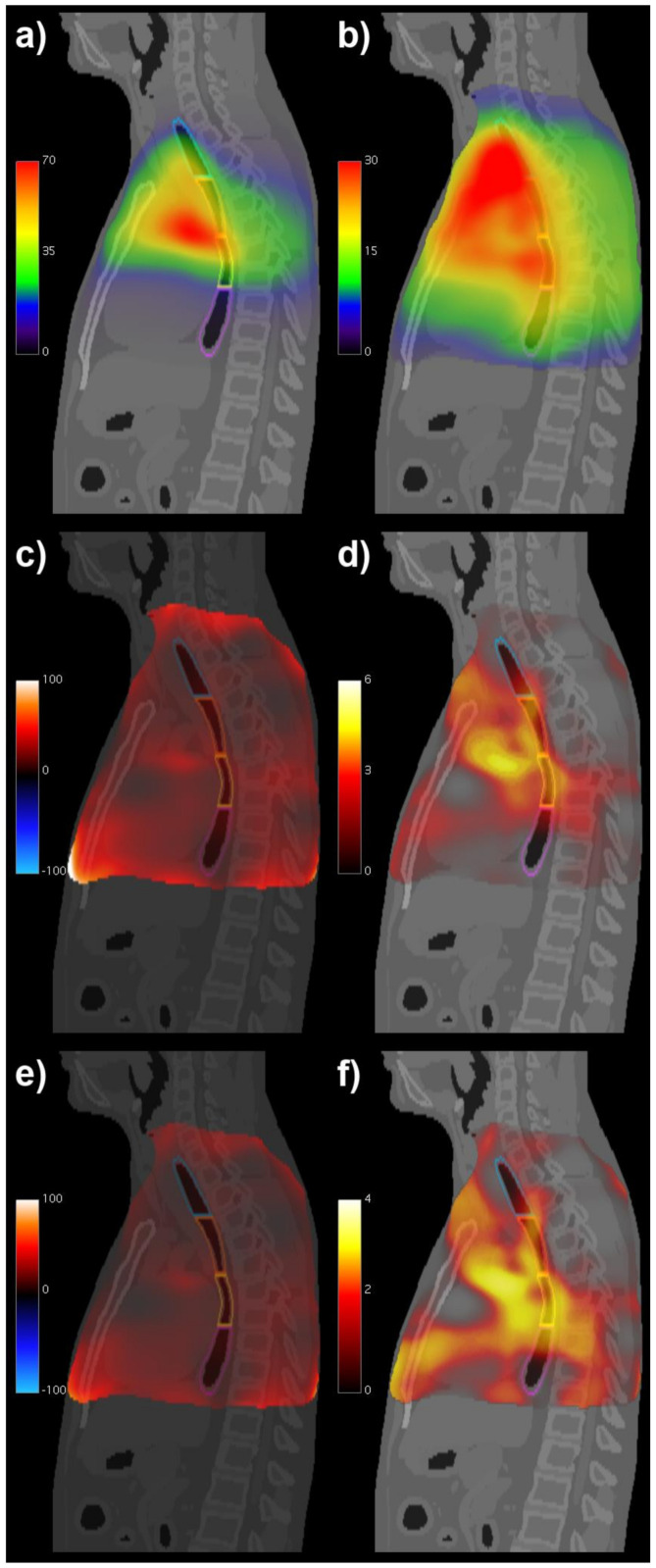
Voxel-based analyses results: sagittal computed tomography (CT) views fused with voxel-wise mean (**a**) and voxel-wise standard deviation (**b**) of biologically effective dose (BED, in Gy) maps; BED coefficient (**c**) and its significance (expressed as -log *p*) (**d**) in the voxel-based GLM of radiation-induced esophagitis (RE), including treatment modality; BED coefficient (**e**) and its significance (expressed as -log *p*) (**f**) in the voxel-based actuarial regression of RE, including induction chemotherapy and treatment modality. The contours of the esophageal subsites are superimposed on the CT images (blue: cervical esophagus, orange: upper thoracic esophagus, yellow: middle thoracic esophagus, and violet: lower thoracic esophagus).

The mean and standard deviation of the BED maps computed voxel by voxel over the patients are displayed in Figure 2a,b. The VBA, designed by including the RT modality as a covariate within the GLM, identified regions of significant association between the BED and RE along a roughly cylindrical region encompassing the thoracic esophagus (Figure 2c,d). The actuarial VBA was performed, including RT and induction CHT as covariates, and confirmed the impact of dose to the same subsites on RE onset as highlighted in Figure 2e,f.

**Figure 3:**
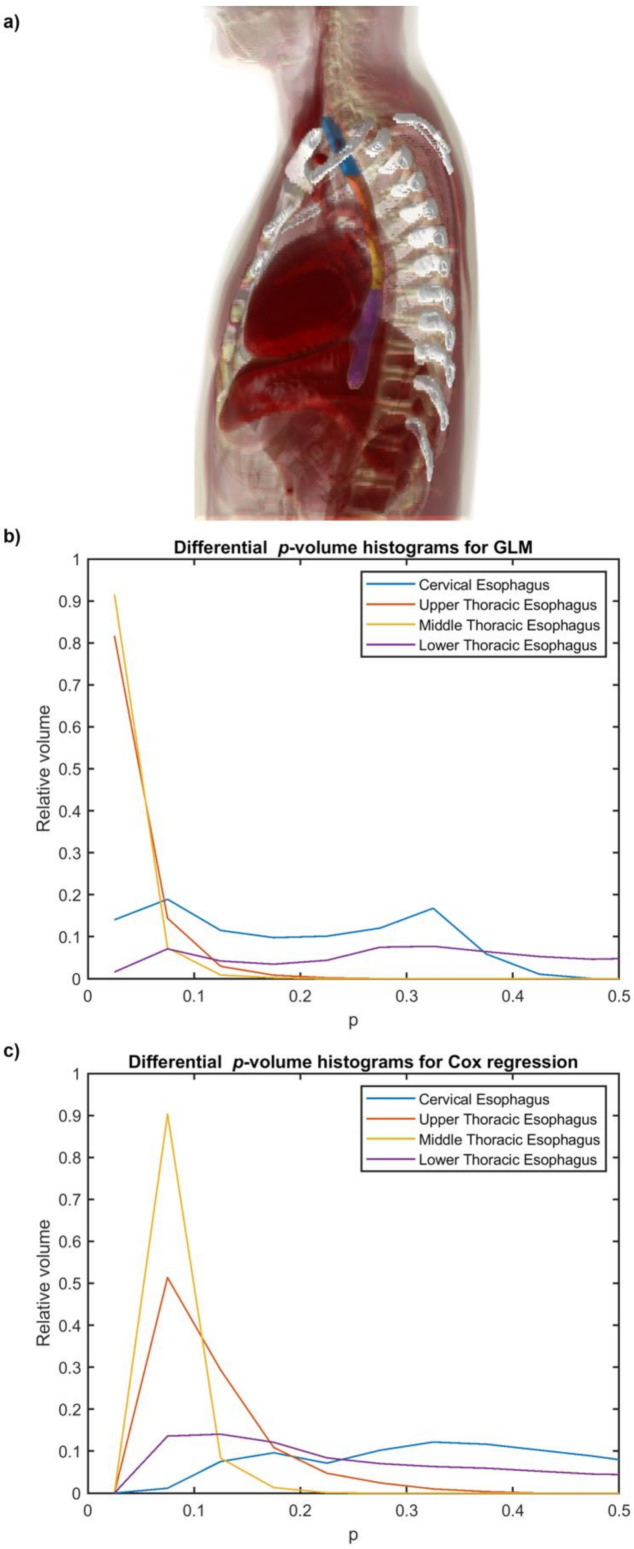
3D volume rendering of the segmented esophageal subsites on the digital phantom (**a**). Relative differential *p*-volume histograms for esophageal subsites according to voxel-based GLM (**b**) and voxel-based actuarial regression (**c**) of RE. Blue: cervical esophagus, orange: upper thoracic esophagus, yellow: middle thoracic esophagus, and violet: lower thoracic esophagus.

The 3D renderings of the segmented esophageal subsites are shown in Figure 3a. In Figure 3b,c, the differential *p*-volume histograms for the esophageal subsites provides a representation of the relative contribution of each subsite to RE development. Notably, the most significant regions matched the upper and middle subsites.

**Figure 4:**
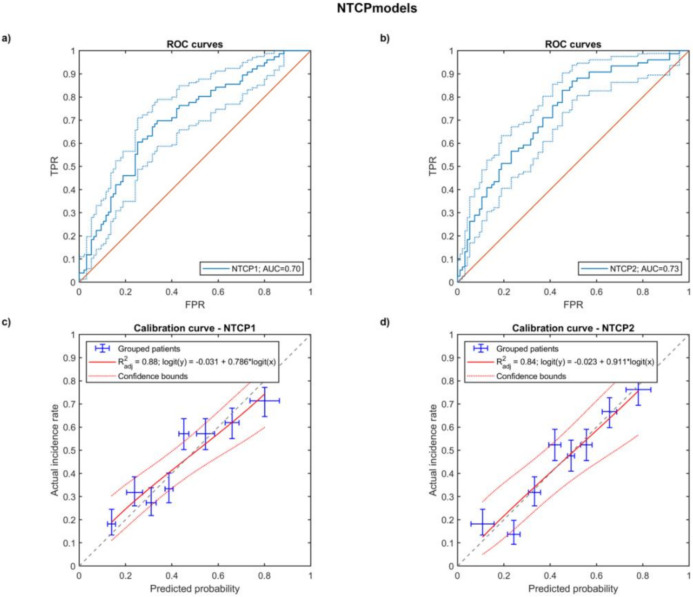
Cross-validated ROC curves of the multivariable logistic regression model (FPR: false positive rate, TPR: true positive rate) for NTCP_1_ (**a**) and NTCP_2_ (**b**); cross-validated calibration plot for NTCP_1_ (**c**) and NTCP_2_ (**d**), the error bars for the reported values represent the 68% confidence intervals.

As for the predictive modeling, we first developed a hierarchical logistic NTCP model (NTCP_1_) using DVH metrics from the entire esophagus. After the variable selection procedure, multivariable modeling resulted in a two-variable model, including RT modality and esophageal V_55Gy_ as predictive factors. On the basis of the VBA findings, a second hierarchical logistic model (NTCP_2_) was developed using DVH metrics extracted from the union of upper and middle thoracic esophageal subsites propagated to the native patient space. The NTCP_2_ model included RT modality and D_mean_ as the most predictive factors. Model parameters and performances are reported in Table 2 and displayed in Figure 4. LOO cross validation confirmed good prediction and calibration performances for both models, with a slightly improved prediction capability by NTCP_2_, compared to NTCP_1_ (CV-AUC = 0.73 and calibration slope = 0.9 vs. CV-AUC = 0.70 and calibration slope = 0.8).

## 4. Discussion

Strengthening our understanding of the radiation physiopathology of normal tissue side-effects may not only lead to an improved prediction, but also to the identification of potential strategies for reducing or preventing toxicity [44,45].

As for the esophagus, a series of molecular mechanisms were recognized to be involved in the RT-induced esophageal injury; the occurrence and severity of acute esophageal toxicity was associated with the subsequent development of late esophagitis, suggesting a partial consequential relationship between acute and late injury [4]. Given the shared pathophysiology and the correlation between acute and late esophagitis, it is expected that prevention of acute RE may reduce delayed effects after RT [46].

However, it is unknown whether the dose–effect relation may be ruled by a markedly inhomogeneous regional response, with the upper part of the esophagus more sensitive to radiation [14,15] due to a longitudinal topographic variation in the sensory nerves [14]. Exploring the spatial patterns of dose–response mechanisms could be particularly useful with the introduction of the most modern treatment modalities, which improve the control over the spatial delivery of the dose to healthy tissues. Additionally, their high therapeutic ratio allows for increasing the attention to the amelioration of quality of life in patients undergoing RT, thus shifting the focus to milder and milder toxicity grades.

In this scenario, with the aim of exploring the potential regional differences of radiation sensitivity in the esophagus, we analyzed the data on esophageal toxicity from a randomized trial on PSPT vs. IMRT treatment for inoperable NSCLC patients. We first applied a VBA approach, followed by a multivariable logistic NTCP modeling strategy.

The observed incidence of acute RE in the analyzed cohort of patients is comparable with data reported in recent studies when concurrent CHT is administered [11,47]). Notably, the group treated with proton therapy showed a higher incidence of RE compared to the group undergoing photon therapy, although, for grade 3 esophagitis, the incidence in the two treatment modality groups was comparable (*p* = 0.136).

As pointed out by Wang et al. [48], although PSPT significantly reduced the volume irradiated at low-dose levels, in the high-dose region, this modality was less conformal than IMRT (Figure 1b). As such, at an organ-based analysis, PSPT might have increased the high-dose volume in the esophagus adjacent to the target, although this behavior does not seem to be significant (Figure 1d).

The RT modality was thus included as a nuisance variable in the performed VBAs accounting for a single time-point as well as for an actuarial toxicity analysis of the RE response to dose in the entire thorax. Interestingly, they both highlighted very similar patterns of dose effects in the regions around the esophagus, which presents an important insight on the self-consistency of the analysis. This bears witness to the quality of VBA inferences in a radiation oncology setting, where the presence of intrinsic spatial autocorrelations of the dose distributions could cast some doubts on the resolution of the VBA results [49].

The regions associated with the most relevant dose effect appear to involve the upper and middle thoracic esophagus (Figure 2c–f and Figure 3b,c). These findings support the pathophysiological pathway to RE hypothesized in previous studies [14,15]. However, it is unclear if the seemingly reduced involvement of the cervical and lower thoracic subsites might be associated with the inhomogeneous patterns of the first two voxel-wise statistical moments of the dose distributions in the analyzed cohort, which suggest a lower statistical power of the VBAs in the margins of the field of view.

According to the obtained VBA results, we compared two different strategies for NTCP modeling. The first one (NTCP_1_) was a multivariable logistic regression based on a statistical selection of both non-dosimetric variables and standard dosimetric variables extracted for the whole esophagus. The second one (NTCP_2_), on the other side, explored as dosimetric variables the DVH metrics extracted for the region composed of the upper and middle thoracic esophagus. Such esophageal subsites were automatically defined by splitting the original esophagus planning contours according to the back propagation in patients’ native space of the subsite boundaries in the common anatomical reference. This procedure allowed both to minimize intra-patient contouring variability of subsites and to avoid changes in the total esophagus volume that could affect NTCP comparisons.

For both NTCP models, we decided to design a hierarchical scheme in order to test if RT modality actually improved the predictive performances of the models based on the only dose metrics. Indeed, the hierarchical process resulted in the inclusion in both models of the RT modality, which thus seems to be a relevant explanatory variable for the RE in addition to the strictly dosimetric variables. The findings of our NTCP analysis were consistent with previous studies examining the esophageal dosimetric predictors of RE. Most studies reported that the high-dose metrics (e.g., dose > 50 Gy) are strongly associated with RE [6]. In particular, in a large individual-patient-data meta-analysis [50], the V_60Gy_ emerged as the best predictor for both moderate and severe RE. On the other hand, it should not surprise us that, when restricting the analysis to an anatomical subsite, the mean dose emerges as the most predictive dosimetric factor.

In this respect, it should be acknowledged that NTCP_2_, while showing good performances at an internal validation, might miss a predictive power in patients with a sensibly different distribution of tumor positions. This aspect is related to two independent points: the previously discussed issue of inhomogeneous patterns of the voxel-wise statistical moments of the dose distributions; and the somehow arbitrary choice of the significance threshold for either including or not a subsite within the spatial domain from which the DVH metrics for NTCP_2_ are extracted. As a result, a test patient with sensibly high doses delivered to the esophagus subsites excluded from the NTCP_2_ computation would likely receive an underestimated RE probability. A proper solution to the issue would deserve the estimation for a non-binary mask of the organ radiosensitivity, following, for instance, the approach proposed in [51]. We aim to explore this strategy of RE modeling on larger cohorts of patients, which could support a robust empirical estimation of the esophagus radiosensitivity.

## 5. Conclusions

We developed two predictive models for acute RE that appear to be substantially equivalent in terms of predictive performances and discrimination capabilities, with a slight improvement provided by the analysis of thoracic esophageal subsites. We suggest that the incremental predictivity is due to the relevant sparing of cervical and lower thoracic esophagus in the analyzed cohort. Further studies on larger cohorts and a more heterogeneous set of dose distributions are required to validate these preliminary findings and shed further light on NTCP modeling that could account for the spatial patterns of RE development [51].

## Figures and Tables

**Table 1:** Patient and treatment characteristics and univariate analyses for radiation-induced esophagitis (RE).

Characteristics	173 pts	*p*-Value *	Cox-*p*-Value
Continuous Variables	Median (Range)		
Age at RT (yr)	66 (33–85)	0.48	0.36
Weight (Kg)	79.3 (47.0–131.0)	0.59	0.89
KPS baseline	80 (60–100)	0.31	0.10
Categorical variables	*N* (%)		
Gender			
Female	77 (44)	0.28	0.35
Male	96 (56)		
Smoker			
No	15 (9)	0.59	0.39
Yes	158 (91)		
Induction chemotherapy			
No	116 (67)	0.073	0.037
Yes	57 (33)		
Adjuvant chemotherapy			
No	132 (76)		
Yes	40 (23)	0.86	0.71
Missing	1 (1)		
RT modality			
IMRT	109 (63)	0.013	0.025
PSPT	64 (37)		
Prescription dose			
66 Gy	68 (39)	0.87	0.57
74 Gy	105 (61)		
Tumor Histology			
Adenocarcinoma	89 (51)		
Squamous cell carcinoma	58 (34)		
NSC NOS	19 (11)	0.53	
Large Cell	3 (2)		
Unknown	4 (2)		
Tumor localization			
Left lung	64 (37)	0.74	0.64
Right lung	109 (63)		
Lower/middle lobe	53 (30)	0.13	0.12
Upper lobe	109 (67)		

Abbreviations. RT: radiation therapy; GTV: gross tumor volume; KPS: Karnofsky performance status. * Mann–Whitney *U* test for continuous variables and Pearson’s χ^2^ test for categorical variables.

**Table 2:** Multivariable logistic regression model coefficients and model performance for radiation-induced esophagitis using the whole esophagus (NTCP_1_) and the thoracic esophagus (NTCP_2_).

NTCP_1_				NTCP_2_			
Model Variables	Coefficient	SE	*p*	Model Variables	Coefficient	SE	*p*
V_55Gy_	5.26	1.14	<0.001	D_mean_ (Gy)	0.06	0.01	<0.001
RT modality	−0.81	0.35	0.02	RT modality	−0.90	0.35	0.01
Constant	−1.18	0.41	0.004	Constant	−2.50	0.69	<0.001
Performance	Value	CI/SE		Performance	Value	CI/SE	
AUC	0.74	[0.66, 0.81]		AUC	0.75	[0.67, 0.81]	
CV-AUC	0.70	[0.62, 0.78]		CV-AUC	0.73	[0.65, 0.80]	
Calibration slope	0.79	0.11		Calibration slope	0.91	0.15	
Calibration intercept	−0.03	0.11		Calibration intercept	−0.02	0.15	

Abbreviations. V_55Gy_: esophageal volume receiving at least 55 Gy; D_mean_: mean dose; RT: radiation therapy; CI: 95% confidence interval; SE: standard error; AUC: area under the Roc curve; and CV: cross validation. RT modality: 0 = PSPT; 1 = IMRT.

## Data Availability

The data presented in this study are not publicly available due to restrictions in the Material Transfer Agreements.

## Supplementary Materials

The following supporting information can be downloaded at: https://www.mdpi.com/article/10.3390/cancers14071833/s1, Figure S1: Cumulative risk of radiation-induced esophagitis (RE) stratified by (a) induction chemotherapy (CHT) and treatment modality (intensity-modulated radiation therapy—IMRT, and passively scattering proton therapy—PSPT); Figure S2: Sagittal computed tomography (CT) views fused with voxel-wise mean of biologically effective dose in patients without (a) and with radiation-induced esophagitis (b); Table S1: Patient and treatment characteristics for the full cohort and for the analyzed one.
