# Supplementary material for: Radiation-Induced Esophagitis in Non-Small-Cell Lung Cancer Patients: Voxel-Based Analysis and NTCP Modeling
Source: Cancers (Basel). 2022 Apr 5;14(7):1833. doi: 10.3390/cancers14071833 (PMC8997452; doi:10.3390/cancers14071833)

Supplementary materials to

Radiation induced esophagitis in non-small cell lung cancer patients: voxel-based analysis and NTCP modeling.

Monti S. *et al.*

Table S1. Patient and treatment characteristics for the full cohort and for the analyzed one.

**Characteristics**	**Full cohort (n=202)**	**Patients with complete CT&dose info (n= 173)**
Continuous variables	**Median (range)**	***Median (range)***
Age at RT (yr)	66 (33-85)	66 (33-85)
Weight (Kg)	78.8 (47.0-153.0)	79.3 (47.0-131.0)
KPS baseline	80 (60-100)	80 (60-100)
Categorical variables	N (%)	***N (%)***
Gender		
Female	92 (45)	77 (44)
Male	110 (55)	96 (56)
Smoker		
No	17 (8)	15 (9)
Yes	185 (92)	158 (91)
Chemotherapy		
Induction		
No	130 (64)	116 (67)
Yes	72 (36)	57 (33)
Adjuvant		
No	157 (77)	132 (76)
Yes	44 (22)	40 (23)
Missing	1 (1)	1 (1)
RT modality		
IMRT	127 (63)	109 (63)
PSPT	75 (37)	64 (37)
Prescription dose		
66 Gy	78 (39)	68 (39)
74 Gy	124 (61)	105 (61)
Tumor Histology		
Adenocarcinoma	107 (53)	89 (51)
Squamous cell carcinoma	69 (34)	58 (34)
NSC NOS	19 (9)	19 (11)
Large Cell	3 (2)	3 (2)
Unknown	4 (2)	4 (2)
Tumor localization		
Left lung	70 (35)	64 (37)
Right lung	119 (59)	109 (63)
Lower/middle lobe	60 (30)	53 (30)
Upper lobe	129 (66)	109 (67)

Abbreviations. RT: Radiation Therapy, GTV: Gross Tumor Volume; KPS: Karnofsky Performance Status; IMRT: intensity modulated radiation therapy; PSPT: passive scattering proton therapy, NSC NOS: non-small-cell not otherwise specified

Figure S1. Cumulative risk of radiation-induced esophagitis (RE) stratified by (a) induction chemotherapy (CHT) and by treatment modality (intensity modulated radiation therapy – IMRT, and passively scattering proton therapy – PSPT).

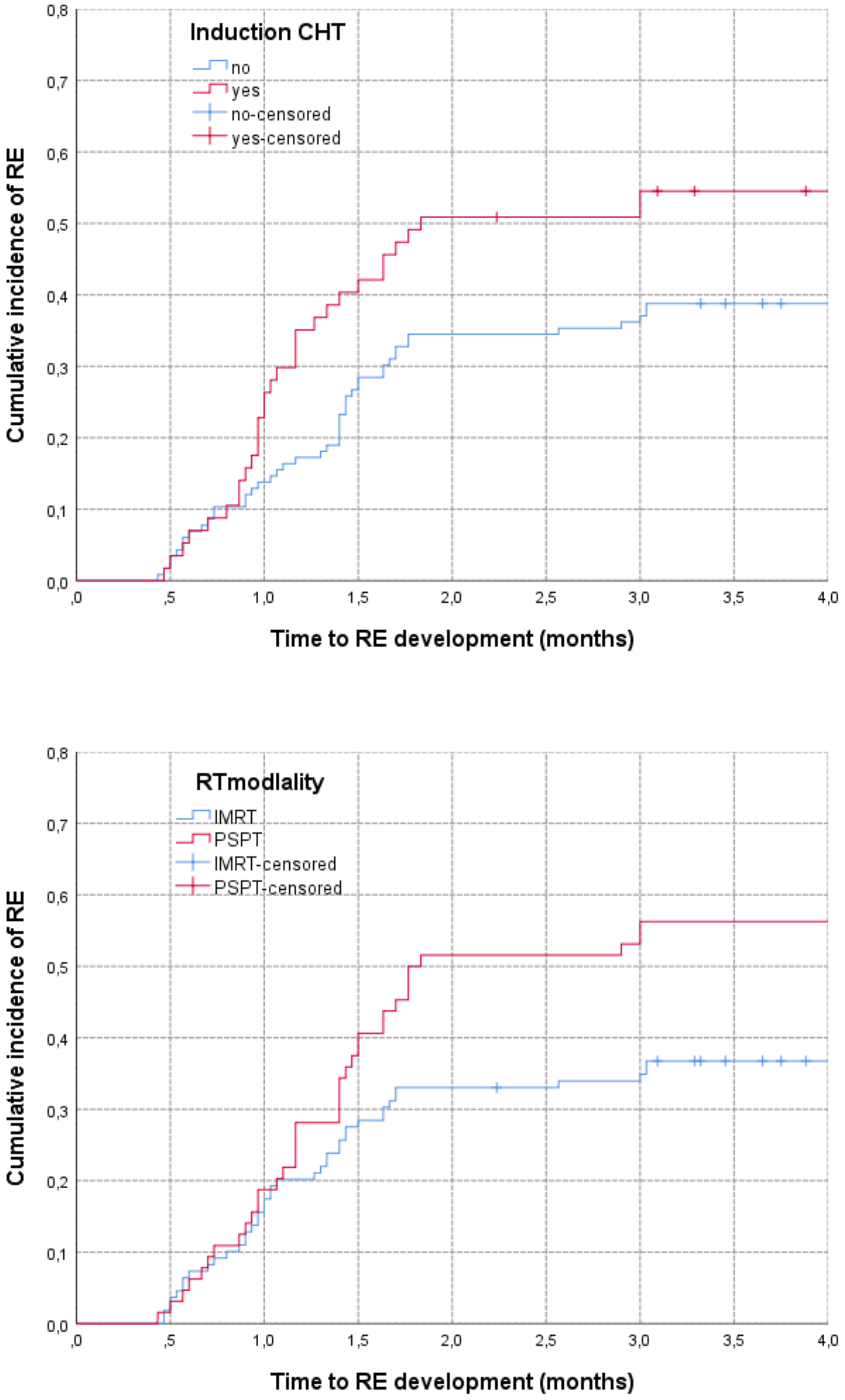

Figure S2. Sagittal computed tomography (CT) views fused with voxel-wise mean of biologically effective dose (BED, in Gy) in patients without (a) and with radiation-induced esophagitis (b)

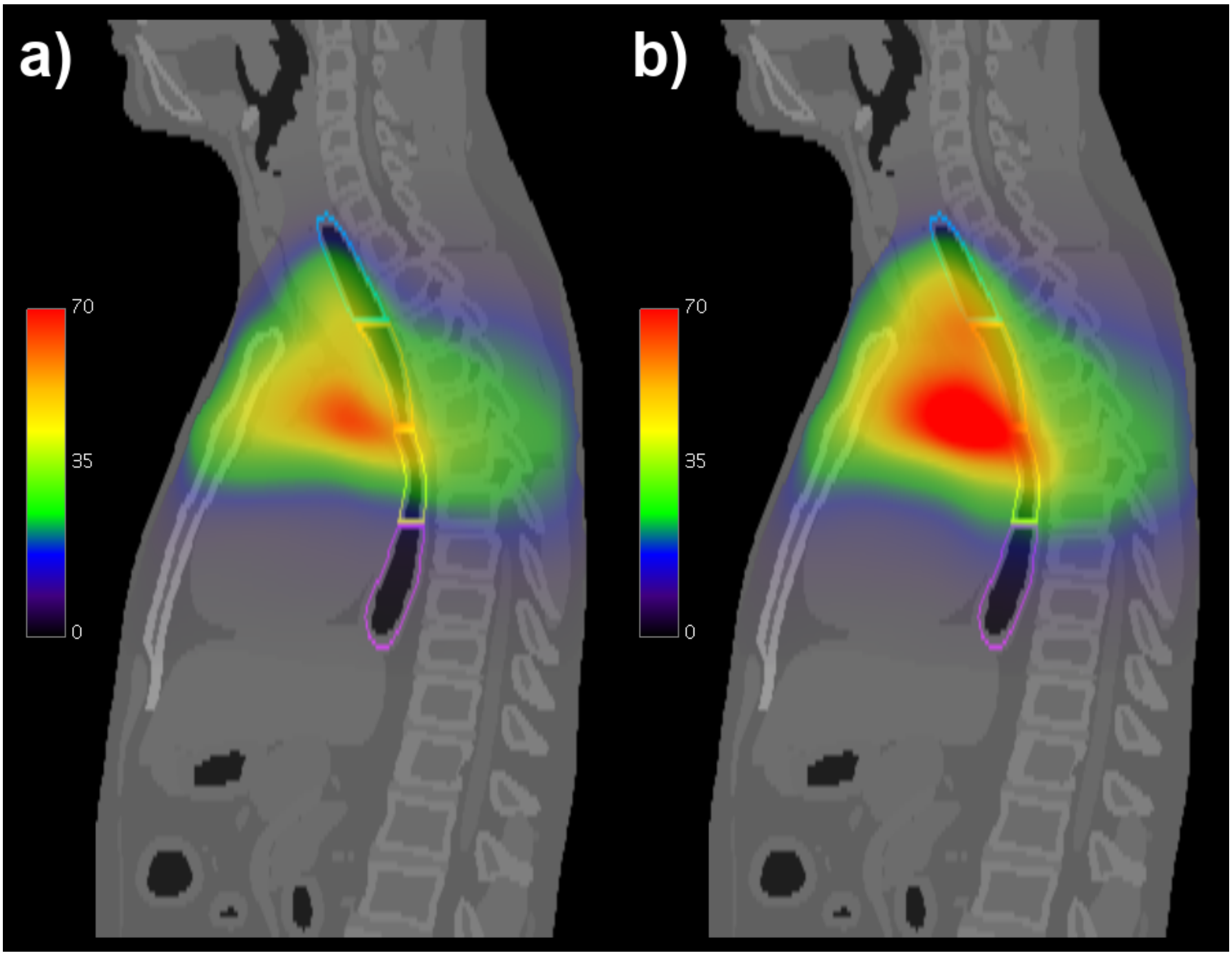

Supplement: Supplementary file 1 [file cancers-14-01833-s001.zip › cancers-1648741-supplementary.pdf]
